# Individual-level transitions between chronic disease multimorbidity clusters and the risk of five-year mortality in longitudinal cohort of Chinese middle-aged and older adults

**DOI:** 10.1007/s40520-025-03078-5

**Published:** 2025-07-09

**Authors:** Xiashan Dong, Yiming Ma, Huizi Zhang, Peigang Wang

**Affiliations:** 1 Department of Social Medicine and Health Management, School of Public Health, Wuhan University, 115 Donghu Road, Wuhan, Hubei China; 2 Center for Population and Health Research, Wuhan University, Wuhan, Hubei China; 3 School of Mathematics, University of Edinburgh, Edinburgh, UK

**Keywords:** Multimorbidity cluster, Additive effect, Compounding effect, Health outcome prediction, Chronic disease

## Abstract

**Background:**

We aimed to trace individual’s transition between multimorbidity clusters and examine the addictive and compounding effects of transition trajectories, chronic disease accumulation, and five-year all-cause mortality.

**Methods:**

Participants from the China Health and Retirement Longitudinal Study (2011–2020) were included (N = 8988). Latent class analyses, Cox proportional hazard models, and restricted cubic splines were used to examine the associations.

**Results:**

Five clusters were identified: osteoarticular, cardiometabolic, multisystem, digestive, and respiratory. Participants who had multisystem multimorbidity and further developed respiratory diseases had mortality risk 9 times higher than the healthy participants (HR:9.04; 95% CI 3.44–23.73). For participants experienced prolonged cardiometabolic multimorbidity, the mortality risk increased by 26% with each additional chronic disease and by 38% with each additional body system affected between 2011 and 2015.

**Conclusion:**

Subsequent interventions should prioritize those who experienced prolonged multi-system multimorbidity, developed respiratory diseases from existing multi-system conditions, or developed additional chronic diseases from existing cardiometabolic multimorbidity.

**Supplementary Information:**

The online version contains supplementary material available at 10.1007/s40520-025-03078-5.

## Introduction

Multimorbidity is defined as the coexistence of two or more chronic diseases within one person [1]. Over half of the global population over 60 years of age are affected by multiple chronic conditions [2–5]. Different combinations of chronic diseases form various clusters of multimorbidity, exhibiting diverse long-term impacts on health outcomes in middle-aged and older adults [6, 7]. Studies indicate that chronic diseases tend to accumulate in specific clusters. Reviews of the literature have also provided insights into these clusters. For instance, a review found 97 patterns of two or more diseases with 63 of these patterns including three or more diseases [8], and another review identified cardiometabolic, musculoskeletal, mental, and respiratory patterns as the most prevalent clusters among 97 studies [9]. Additionally, patterns involving two or three defining diseases had a higher incidence in middle-aged and older adults, with the complexity of disease combinations increasing with age [10]. However, although there has been well-established evidence of associations between multimorbidity and lower quality of life [11], depression and polypharmacy [12–14], and shorter life expectancy [15], minimal emphasis has been put on the individual-centred transition between these multimorbidity clusters (longitudinal cluster combinations) over time [16, 17]. Overlooking such additive effects of multimorbidity clusters could underestimate the adverse impact of multimorbidity development, as well as the compounding effect of chronic disease accumulation. This study focused on middle-aged and older adults to examine how transitions between multimorbidity clusters shape mortality risk in aging populations.

Current research on multimorbidity clusters has evolved along two primary axes: disease-centered clustering and individual-centered clustering. The former axe includes methods such as association rule mining, network analysis, etc., focusing on identifying prevalent disease co-occurrence patterns at the population level that could reveal epidemiological trends [18, 19]. The individual-centered methods often classify participants into subgroups based on their unique disease profiles, enabling investigations into health outcome heterogeneity. Widely used techniques in previous studies include K-means, fuzzy c-means, latent class analysis (LCA), etc., or longitudinal techniques such as latent transition analysis or latent class growth analysis [20–22]. In this study, we repeatedly applied LCA to model the multimorbidity clusters from 14 diseases (coded as binary variables) across three waves. Such method selection considered that while longitudinal methods like latent transition analysis (LTA) or multistate models could theoretically track transitions, their computational complexity risks overfitting and obscuring clinically interpretable phenotypes. By contrast, repeated LCA at each wave allows us to prioritize dynamic shifts between stable, interpretable clusters—an approach validated in prior aging cohort studies also using similar soft clustering technique[23].

Individual transitions between multimorbidity clusters may result from the development of key diseases in other clusters triggered by existing conditions or iatrogenic events. Therefore, tracking these transition trajectories and examining the additive effects of cluster combinations over time provide an efficient way to assess disease development in a large population. Although some studies have examined disease combinations within individuals, they are often limited to high-incidence disease groups, such as cardiometabolic disease [24], without considering the non-random associations and broader context of multimorbidity clusters affecting multiple body systems. While some research has touched on the individual's trajectory over time descriptively [23], they have not associated the long-term transitions with adverse health outcomes statistically, which motivates us to conduct this research. Further, the majority of the evidence were from the Western population instead of the Chinese population. Therefore, in this study, we traced the individuals’ longitudinal movement between multimorbidity clusters and aimed to examine the longitudinal additive effects of multimorbidity clusters and the association between cluster combinations and five-year all-cause mortality risks. In addition, within each cluster combination, we further assessed the influence of chronic disease accumulation and the number of affected body systems on mortality.

## Material and methods

### Data source and sample collection

The China Health and Retirement Longitudinal Study (CHARLS) is a nationwide longitudinal study of middle-aged and older adults in China. Participants enrolled in CHARLS were randomly selected using a stratified, multistage sampling strategy. After the initial survey between 2011 and 2012, CHARLS conducted four follow-up surveys in 2013, 2015, 2018 and 2020. CHARLS was approved by the Ethics Committee of Peking University Health Science Center and informed consent was obtained from all participants. Detailed sampling methodology, protocols, and data descriptions have been reported previously [25, 26].

In this study, data for a total of 25,586 participants were derived from the five waves of surveys. Participants were excluded from the analysis if they: 1) do not have complete data for all three waves from 2011 to 2015 ($n=$ 12,021); 2) have missing values of chronic disease conditions for at least one wave from 2011 to 2015 ($n$=30); 3) have missing values of covariates for at least two waves ($n=$ 3,041).

Our primary analysis focused on individuals with at least one chronic condition in 2011 to examine transitions between multimorbidity clusters. However, to contextualize mortality risks, we included a reference group of participants who reported no chronic diseases mentioned above across all three waves (2011, 2013, 2015). This group, labelled as “no-NCD counterparts” served to benchmark mortality outcomes against individuals with identified multimorbidity clusters (eTable 2). While this definition excludes undiagnosed or subclinical conditions, it ensures comparability in analyzing transitions among pre-existing multimorbidity clusters. The flowchart of the sample selection process is shown in eFigure 1. A total of 8988 participants were finally included for the subsequent analysis, with 7144 participants with chronic diseases in the first three waves and 1844 no-NCD counterparts to serve as the reference level in the Cox regression analysis.

### Measures

#### Chronic conditions and multimorbidity

In this study, we defined multimorbidity as the coexistence of two or more chronic conditions within one person [27, 28]. We ascertained 14 chronic disease conditions based on a combination of biomarker information (hypertension, defined by systolic/diastolic blood pressure ≥ 140/90 mmHg) and self-reported physician diagnosis (diabetes or high blood sugar; cancer or a malignant tumour [excluding minor skin cancers]; chronic lung disease; heart problems; stroke; emotional, nervous, or psychiatric problems; arthritis or rheumatism; dyslipidemia; liver disease [except fatty liver]; kidney disease; digestive disease; asthma; and memory-related disorders). Participant’s historical chronic conditions were assessed at three waves (2011, 2013, 2015) to capture multimorbidity transitions while balancing analytical complexity and statistical power. Participants were asked to confirm the chronic diseases or conditions reported in the previous round in each follow-up survey of the three waves to ensure data accuracy. All diseases and conditions were assumed to persist throughout the follow-up period once they were identified, and they were coded as binary variables for further identification of multimorbidity patterns.

#### Mortality

The primary outcome was death from all causes, identified through reports from the participant’s next of kin in wave 2018 and wave 2020. Valid information regarding the mortality status was derived from two types of variables provided by CHARLS: a binary variable indicating whether a participant responded to each survey wave and a categorical variable representing their mortality status. To refine survival time calculations, the exact date of death was obtained from the 2020 wave. Survival time was calculated from the end of the multimorbidity pattern assessment (2015 wave) until the reported death year or censoring (dropout/last follow-up). This design prioritizes clinically interpretable 5-year mortality risk (2015–2020) while avoiding overcomplication from irregular intervals.

#### Covariates

Sociodemographic and lifestyle factors that may account for the progression of multimorbidity were used as covariates in this study. These factors included age (categorized as 45–64 and 65 +), sex (male or female), education level (no formal education, elementary school, middle school or above), marital status (married/partnered or unmarried/unpartnered), smoking status (non-smoker or smoker), and alcohol consumption (non-drinker or drinker). Smoking status (current smoker: yes/no) and alcohol consumption (current drinker: yes/no) were defined based on self-reported tobacco use and alcohol intake in the past year at baseline. Particularly, Hukou status (the household registration system in China) was selected as a proxy for systemic disparities in healthcare access, as it is closely tied to eligibility for medical and public health insurance programs and the accessibility of medical resources in China [29, 30]. These variables were selected based on their established roles in capturing socioeconomic disparities and health inequities in multimorbidity research [9, 31]. Additionally, the number of chronic diseases and the number of affected body systems at each follow-up wave were included to account for the cumulative effects among multimorbidity clusters. Chronic diseases and their corresponding systematic classifications are detailed in eTable 1.

### Statistical analysis

Latent class analysis (LCA) was initially conducted to identify participants sharing latent multimorbidity patterns across the first three waves of the survey. LCA does not require the specification of any distance measurement, making it superior to other clustering algorithms when analyzing participants’ responses to categorical indicator variables [32]. To determine the optimal number of clusters (chosen between 3 and 10), a sequence of LCA models were constructed separately for each of the first three survey waves. For each candidate number of clusters, the model was repeated 100 times to provide average final solutions. The optimal number of classes was selected by comparing the Akaike Information Criteria (AIC), Bayesian Information Criteria (BIC), Chi-square statistics, and G-square statistics. To characterize each multimorbidity cluster, observed/expected ratios (O/E ratios) and disease exclusivity rates were calculated, acknowledging the possibility of participants developing common diseases across different clusters. A disease was considered a defining feature within a cluster if the O/E ratio $\ge$ 2 or exclusivity rates $\ge$ 0.25, with both criteria satisfied for naming the cluster [21, 23]. Variables were categorized and summarized using frequencies and percentages. The Pearson ${\upchi }^{2}$ test and one-way analysis of variance were employed to explore group disparities.

Hazard ratios (HRs) and 95% confidence intervals (CIs) were calculated using Cox proportional hazard models to assess the association between multimorbidity clusters, transition patterns, and all-cause mortality. The models were adjusted by age, gender, education level, marital status, urban versus rural areas, smoking status, and alcohol consumption. The top 40 multimorbidity transition combinations were selected out of 83 combinations observed from 2011 to 2015. These 40 combinations represented 97.76% of the total population, ensuring adequate sample representation. Afterwards, the cumulative effects of chronic condition count and the number of affected body systems on all-cause mortality were investigated based on multimorbidity transition combinations. All Cox models were examined for the proportional-hazards assumption using the Kolmogorov-type supremum test and found no trend with time. Additionally, we investigated linear and potential nonlinear trends between the number of chronic conditions and affected body systems in 2011, 2015 and HRs (95% CI) for five-year all-cause mortality using restricted cubic spline (RCS) regression.

Multiple chain imputations were performed prior to conducting the Cox proportional regression. To address potential biases from missing data, unmeasured confounders, and covariate adjustment, we implemented the following sensitivity analyses: 1) we repeated the analyses on complete data without multiple chain imputation to confirm consistency with imputed data; 2) we incorporated additional lifestyle confounder as covariate to ensure model stability (binary physical activity variable categorized as whether the participant engaged in any levels of physical activity for at least 10 min per week). Statistical analyses were performed using SAS 9.4 and R (Version 4.2.3), with a two-sided *P* < 0.05 considered statistically significant.

## Results

### Baseline characteristics and multimorbidity clusters

A total of 8988 participants (corresponding to 43416 person-years) aged 44 to 102 were included in this study, where 7144 participants had at least one chronic condition from 2011 to 2015 and 1844 no-NCD counterparts. Among those with chronic conditions, 61.7% were aged 45 to 65, 53.1% were male, and the majority resided in rural areas (74.7%). At baseline, the highest incidences were observed for arthritis or rheumatism (60.4%), hypertension (52.9%), and stomach or other digestive disease (42.9%; Table 1). Compared to participants without the 14 chronic conditions, those with at least one were more likely to be older, female, and had lower education levels (eTable 2). We further compared statistical characteristics of included participants with those excluded due to incomplete follow-up (eTable 3). Excluded individuals with baseline chronic conditions were younger (mean age 58.1 vs. 59.5 years) and had fewer baseline chronic diseases (median 0 vs. 2), suggesting attrition of younger, less severely ill individuals. Conversely, excluded healthy participants were older (57.5 vs. 56.6 years) and less educated (≥ middle school: 33% vs. 38%, *P* < 0.001), indicating underrepresentation of socioeconomically vulnerable subgroups.

**Table 1:** Characteristics of participants with chronic conditions and prevalence of chronic disease in 2015

Characteristics	Total	Arth	Cardm	Compx	Digst	Resp	*P* value
	*n* (%)						
Participants	7144	1285	2237	647	2164	811	-
Age (years)							< 0.001
≤ 65	4405(61.7)	824(64.1)	1319(59.0)	336(51.9)	1516(70.1)	410(50.6)	
> 65	2739(38.3)	461(35.9)	918(41.0)	311(48.1)	648(29.9)	401(49.4)	
Sex							< 0.001
Male	3354(46.9)	582(45.3)	1047(46.8)	279(43.1)	951(43.9)	495(61.0)	
Female	3790(53.1)	703(54.7)	1190(53.2)	368(56.9)	1213(56.1)	316(39.0)	
Hukou Regions							< 0.001
Rural	5333(74.7)	1058(82.3)	1478(66.1)	456(70.5)	1703(78.7)	638(78.7)	
Urban	1811(25.3)	227(17.7)	759(33.9)	191(29.5)	460(21.3)	172(21.3)	
Education							< 0.001
Illiterate	2060(28.8)	440(34.3)	572(25.6)	194(30.0)	636(29.4)	218(26.9)	
≤ Elementary school	2943(41.2)	535(41.6)	846(37.8)	279(43.1)	901(41.6)	382(47.1)	
≥ Middle school	2141(30.0)	310(24.1)	819(36.6)	174(26.9)	627(29.0)	211(26.0)	
Marital status							< 0.001
Married/partnered	5963(83.5)	1065(82.9)	1858(83.1)	521(80.5)	1865(86.2)	654(80.6)	
Unmarried/unpartnered	1181(16.5)	220(17.1)	379(16.9)	126(19.5)	299(13.8)	157(19.4)	
Smoking status							< 0.001
Current smoker	1874(26.2)	354(27.5)	492(22.0)	145(22.4)	626(28.9)	256(31.6)	
None-smoker	5270(73.8)	931(72.5)	1745(78.0)	502(77.6)	1538(71.1)	554(68.4)	
Alcohol consumption							< 0.001
Drink alcohol last year	2229(31.2)	461(35.9)	645(28.8)	163(25.2)	701(32.4)	259(31.9)	
No alcohol last year	4915(68.8)	824(64.1)	1592(71.2)	484(74.8)	1463(67.6)	552(68.1)	
Disease							
Hypertension	3296(52.9)	337(27.0)	1768(79.6)	502(79.1)	446(21.2)	243(30.6)	< 0.001
Diabetes/high blood sugar	958(13.8)	44(3.5)	581(26.7)	229(36.4)	80(3.8)	24(3.0)	< 0.001
Cancer or malignant tumor	157(2.2)	10(0.8)	60(2.7)	35(5.8)	49(2.3)	3(0.4)	< 0.001
Chronic lung disease	1454(20.7)	5(0.4)	81(3.7)	310(48.5)	283(13.3)	775(95.7)	< 0.001
Heart problems	1802(25.8)	60(4.8)	703(32.1)	491(77.1)	349(16.5)	199(25.0)	< 0.001
Stroke	429(6.1)	49(3.8)	245(11.1)	113(17.6)	10(0.5)	12(1.5)	< 0.001
Emotional/nervous/psychiatric problem	257(3.7)	34(2.7)	45(2.0)	64(10.0)	90(4.2)	24(3.0)	< 0.001
Arthritis or rheumatism	4262(60.4)	1285(100)	676(30.8)	558(87.1)	1361(63.9)	382(47.9)	< 0.001
Dyslipidemia	1661(24.4)	1(0.1)	1014(47.1)	380(61.6)	219(10.6)	47(6.1)	< 0.001
Liver disease	659(9.4)	30(2.4)	124(5.7)	191(29.9)	252(11.9)	62(7.8)	< 0.001
Kidney disease	985(14.1)	106(8.4)	167(7.6)	292(46.1)	344(16.2)	76(9.6)	< 0.001
Stomach or other digestive disease	3022(42.9)	49(3.9)	271(12.4)	509(79.4)	2033(94.3)	160(20.1)	< 0.001
Asthma	646(9.2)	57(4.5)	30(1.4)	166(25.8)	11(0.5)	382(47.7)	< 0.001
Memory-related disease	310(4.4)	33(2.6)	148(6.7)	95(14.8)	17(0.8)	17(2.1)	< 0.001
Mortality							
2018	467(7.0)	67(5.5)	153(7.4)	77(12.8)	78(3.8)	92(12.1)	
2020	804(12.1)	117(9.6)	266(12.9)	128(22.1)	142(6.9)	151(20.0)	

Participants were divided into five patterns based on conditions at baseline: osteoarticular multimorbidity (“Arth”), cardiometabolic multimorbidity (“Cardm”), multisystem complex pattern (“Compx”), digestive multimorbidity (“Digst”), and respiratory multimorbidity (“Resp”)

Elbow plots of the model fit evaluation metrics revealed candidate models of 4, 5 and 6 clusters. For each candidate model, diseases’ O/E ratios and exclusivity rates were calculated in every cluster across each wave to determine defining features. Candidate model with five clusters was determined as the optimal with the most reasonable clinical interpretability (eTable 4–6), yielding the following groups (eTable 7–12): osteoarticular multimorbidity (referred to as “Arth” in the following section), cardiometabolic multimorbidity (“Cardm”), multisystem complex pattern (“Compx”), digestive multimorbidity (“Digst”), and respiratory multimorbidity (“Resp”). The baseline characteristics by patterns are detailed in Table 1. The “Compx” group exhibited a higher mortality rate than the other groups in every year of follow-up. The “Cardm” group had the highest proportion of urban residents (33.9%) and educational attainment (36.6%), the “Arth” group had the highest proportion of rural residents (82.3%), and the “Digst” group displayed the highest proportion of smokers (28.9%) and drinkers (32.4%).

**Fig. 1:**
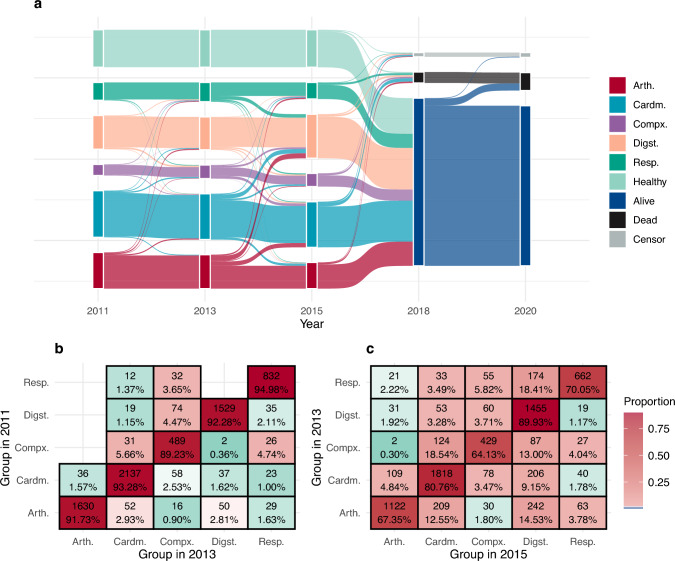
Progression trajectory of older adults with multimorbidity (**a**) and the proportion of individuals transferred between clusters (**b**). Five multimorbidity clusters were named as osteoarticular multimorbidity (“Arth”), cardiometabolic multimorbidity (“Cardm”), multisystem complex pattern (“Compx”), digestive multimorbidity (“Digst”), and respiratory multimorbidity (“Resp”). For (**a**), the height of each box and the thickness of the connecting stripes in the figure are proportional to the cluster sizes and the number of individuals migrating from each cluster, respectively. *Note:* Participants were divided into five patterns based on conditions at baseline: osteoarticular multimorbidity (“Arth”), cardiometabolic multimorbidity (“Cardm”), multisystem complex pattern (“Compx”), digestive multimorbidity (“Digst”), and respiratory multimorbidity (“Resp”)

The transition trajectories between multimorbidity clusters were identified over time. Figure 1a depicts the longitudinal transition from 2011 to 2020. Figure 1b further details participant transitions between clusters across study years. Among the participants with chronic conditions, 27.8% experienced at least one transition across the survey waves, indicating the development of at least one defining disease from other multimorbidity patterns. During the first (2011) and second (2013) follow-up periods, over 90% of individuals in each cluster remained within the cluster in the subsequent period, indicating a degree of short-term stability in multimorbidity patterns. As time progressed, participants shifted toward the “Compx” and “Cardm” groups, indicating that these patterns may be associated with higher health risks and poorer outcomes. In contrast, the “Digest” patterns appeared relatively stable, with 89.9% of the participants staying in the original cluster.

### Association of transition between clusters and all-cause mortality

**Fig. 2:**
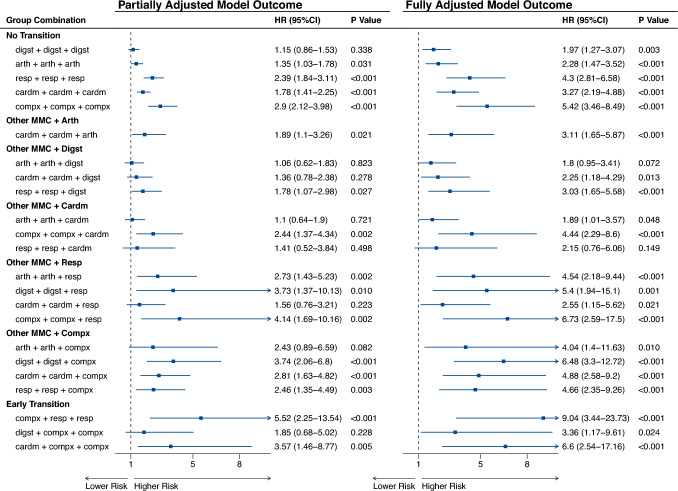
Associations between multimorbidity cluster progression and all-cause mortality risk among middle-aged and older Chinese adults. *Note:* Participants were divided into five patterns based on conditions at baseline: osteoarticular multimorbidity (“Arth”), cardiometabolic multimorbidity (“Cardm”), multisystem complex pattern (“Compx”), digestive multimorbidity (“Digst”), and respiratory multimorbidity (“Resp”).Partially-adjusted model was adjusted for age and gender. Fully-adjusted model was adjusted for all controllers. *P < 0.1, **P < 0.05, ***P < 0.001

Analyses of longitudinal combinations of multimorbidity clusters indicated that transitions from other clusters to the “Compx” and “Resp” clusters have the most substantial impact on elevating mortality risk comparing to the benchmark no-NCD group as the reference (Fig. 2). Among the 40 most prevalent multimorbidity cluster combinations, those involving the “Compx” and “Resp” clusters yielded the highest mortality risk (Compx + Compx + Resp, HR 6.73 [2.59, 17.50]) comparing to no-NCD group. Furthermore, participants initially in the “Compx” cluster who developed respiratory multimorbidity within a short timeframe exhibited mortality risks nearly 10 times higher than no-NCD individuals (Compx + Resp + Resp, HR 9.04 [3.44, 23.73]). While direct empirical evidence for longitudinal respiratory transitions in multisystem multimorbidity remains sparse, indirect support emerges from studies highlighting the clinical vulnerability and mortality risks related to respiratory diseases[33, 34]. Further, pre-existing multimorbidity conditions have been shown to worsen respiratory disease prognoses[35], suggesting a plausible mechanism for the observed risk escalation. This risk elevation likely reflects the compounding burden of multisystem frailty and acute respiratory complications (e.g., COPD exacerbations in patients with preexisting cardiovascular-metabolic dysfunction)[36]. However, the small subgroup size (n = 17) may reduce the statistical power to estimate this association robustly. Notably, earlier transitions (e.g., developing respiratory diseases within the first follow-up interval) were associated with higher mortality than later transitions. In contrast, transitions to the “Digst” or “Arth” groups at the third follow-up wave were associated with lower mortality risks compared to other cluster combinations (Cardm + Cardm + Digst, HR 2.25 [1.18, 4.29]; Cardm + Cardm + Arth, HR 3.11 [1.65, 5.87]). For individuals remaining in the same cluster over five years, mortality risk ranked highest in the “Compx” group (HR 5.42 [3.46, 8.49]), followed by “Resp” (HR 4.30 [2.81, 6.58]), “Cardm” (HR 3.27 [2.19, 4.88]), “Arth” (HR 2.28 [1.47, 3.52]), and “Digst” groups (HR 1.97 [1.27, 3.07]).

### Effects of cumulative characteristics on all-cause mortality

**Fig. 3:**
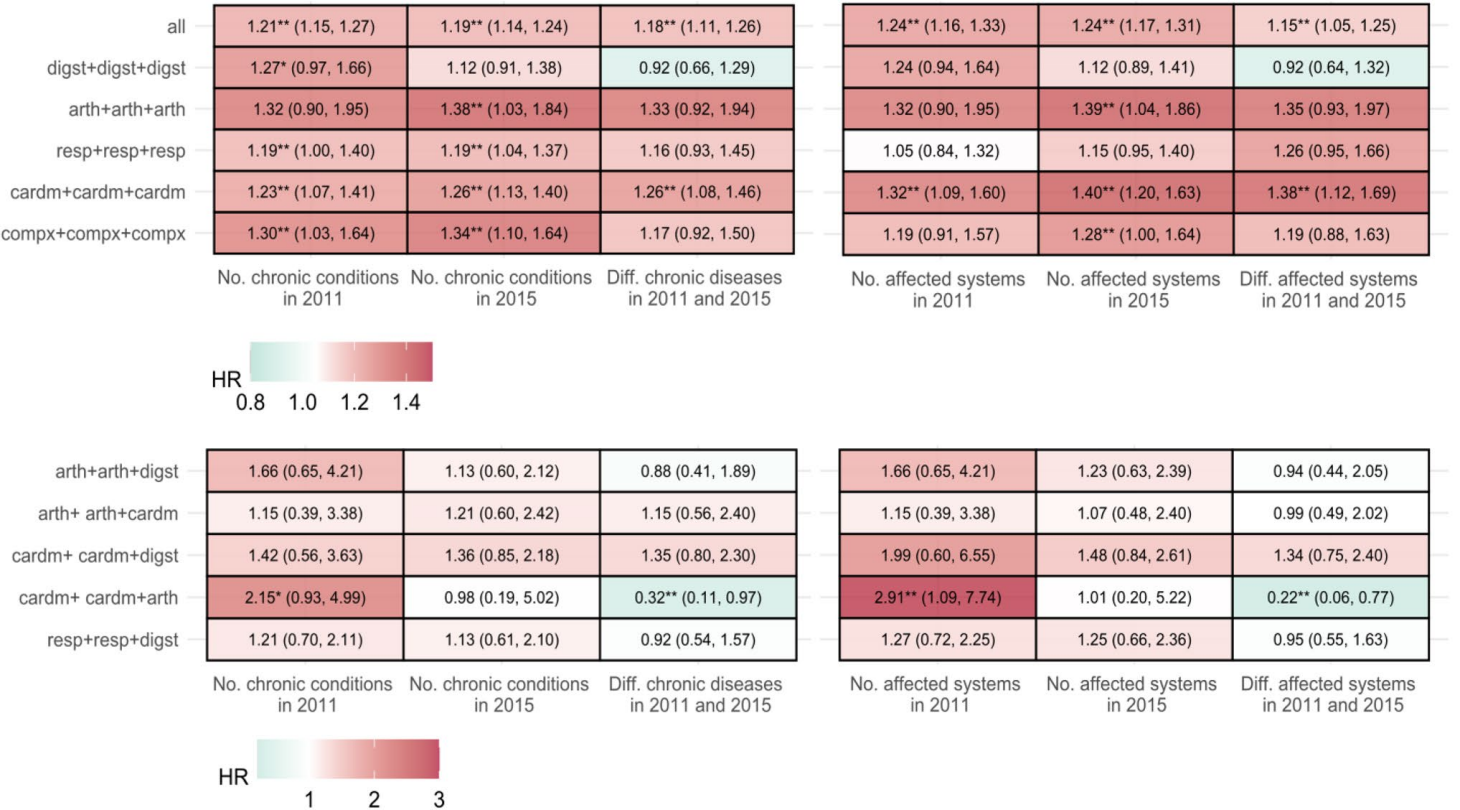
Heat maps based on the associations (hazard ratios) of the cumulative characteristics and multimorbidity pattern combination with the outcomes. *Note:* Participants were divided into five patterns based on conditions at baseline: osteoarticular multimorbidity (“Arth”), cardiometabolic multimorbidity (“Cardm”), multisystem complex pattern (“Compx”), digestive multimorbidity (“Digst”), and respiratory multimorbidity (“Resp”). *P < 0.1, **P < 0.05, ***P < 0.001

Cox regression was employed to assess the association between mortality and combinations of multimorbidity clusters along with cumulative characteristics of chronic diseases. The results indicated significant compounding effects on mortality risk as both the number of chronic conditions and affected systems increased (Fig. 3). For individuals who consistently remained in the “Cardm” and “Compx” groups, cumulative characteristics of chronic diseases—regardless of whether in the early (2011) or advanced (2015) stage—significantly influenced mortality risk. Notably, the number of affected systems had a greater impact on the “Cardm” group (HR 1.40 [1.20–1.63]), while individuals in the “Compx” group were more sensitive to the number of diseases (HR: 1.34 [1.10, 1.64]). The growth rates of disease count and the number of affected systems from 2011 to 2015 were significantly associated with mortality risk for those remaining in the “Cardm” group, with hazard ratios of 1.26 (95% CI 1.08–1.46) and 1.38 (95% CI 1.12–1.69). For individuals initially in the “Cardm” group and later transitioned to the “Arth” group, an increase in the number of affected systems during the early stage was associated with a significantly heightened mortality risk (HR: 2.91 [1.09, 7.74]).

**Fig. 4:**
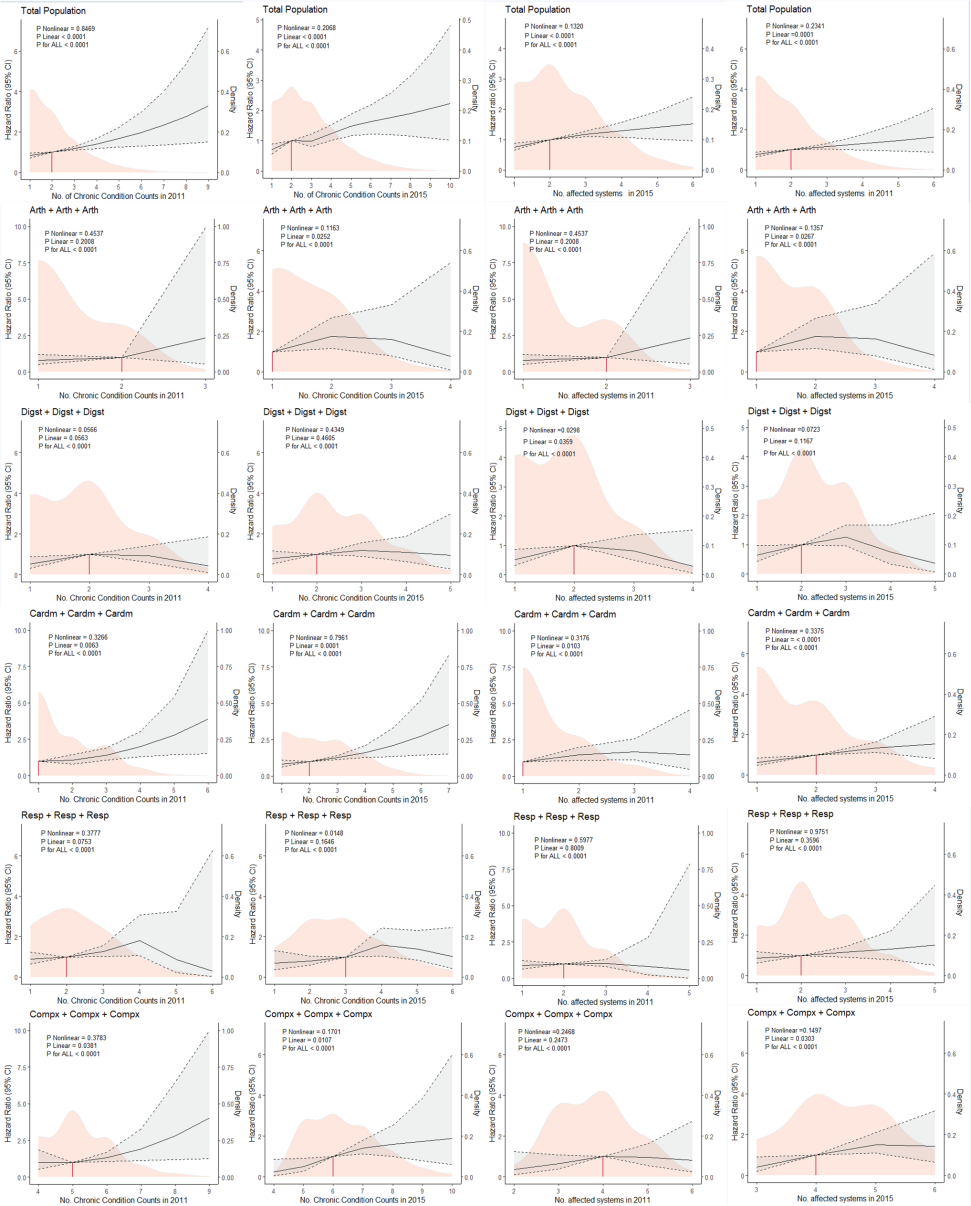
Associations between chronic disease cumulative characteristics and all-cause mortality by multimorbidity transition trajectory among middle-aged and older Chinese adults. *Note:* Participants were divided into five patterns based on conditions at baseline: osteoarticular multimorbidity (“Arth”), cardiometabolic multimorbidity (“Cardm”), multisystem complex pattern (“Compx”), digestive multimorbidity (“Digst”), and respiratory multimorbidity (“Resp”). Partially-adjusted model was adjusted for age and gender. Solid black line: Estimated hazard ratio (HR) with 95% confidence interval (CI) shaded in gray. Orange density curve: Distribution of the predictor variable in the study population. Fully-adjusted model was adjusted for all controllers. *P < 0.1, **P < 0.05, ***P < 0.001

Furthermore, linear-dose–response relationships were observed between the chronic condition counts (*P* for non-linear in 2011: 0.847; in 2015: 0.207), affected body systems (*P* for non-linear in 2011: 0.132; in 2015: 0.234), and growth rate in chronic condition counts between 2011 and 2015 (*P* for non-linear: 0.634; Fig. 4; eFigure 2). The increasing trend in mortality risk plateaued when the number of affected body systems reached a threshold of 2. Significant non-linear relationships were also noted between chronic condition counts (*P* for non-linear in 2011: 0.067) and affected systems (*P* for non-linear in 2011: 0.029; in 2015: 0.072) in the “Digst + Digst + Digst” group, and between differences in chronic condition counts in “Cardm + Cardm + Cardm” group (*P* for non-linear: 0.007; Fig. 4, eFigure 2). This phenomenon may reflect interactions between diseases and adaptive physiological adjustments in patients.

Our findings remained robust across analyses without imputation. We reran all analyses using individuals with complete data or adjusting for additional lifestyle confounder (physical activity). The results remained consistent with our primary findings (eFigure 3, eFigure4), suggesting that our primary models are robust.

## Discussion

To the best of our knowledge, this is the first population-based cohort study to investigate the association between individuals’ transitions between multimorbidity clusters, the cumulative characteristics of chronic diseases—including the number of early and advanced chronic diseases, the number of affected body systems, and the cumulative rate of chronic diseases—and five-year all-cause mortality. Our analyses yielded three main findings, confirming the differential effects of chronic disease characteristics across various transition combinations. First, the way individuals’ transit between multimorbidity clusters exhibited both stability and increasing complexity, suggesting that individuals are increasingly susceptible to complex disease patterns and functional frailty as they age and accumulate additional diseases. Those who transitioned were more likely to move towards complex disease clusters characterized by “Compx” and “Cardm” in the short term, and to “Cardm” and “Digst” in the long term. Second, we confirmed the significant additive effects of multimorbidity clusters on all-cause mortality. Individuals who transitioned to the “Compx” and “Resp” clusters from other clusters significantly increased mortality risk, and those who transitioned from the “Compx” cluster to “Resp” cluster at an early stage had five-year mortality risks nearly 10 times higher than their healthy counterparts. Third, we identified a compounding effect of cumulative chronic diseases on mortality risk within multimorbidity cluster combinations. Overall, the accumulation of chronic conditions and the number of affected body systems were significantly associated with elevated mortality risk, and such associations were particularly significant for participants who were consistently remained in cardiometabolic multimorbidity cluster. Additionally, we found varying effects of cumulative chronic conditions across different trajectories, revealing linear or non-linear dose–response relationships suggesting that mortality risk escalates disproportionately as disease burden increases. While these patterns may hint at underlying biological complexity, our study serves as unique observational evidence awaiting further confirmation of underlying systemic pathways (e.g., chronic inflammation).

### Identify the transition trajectories between multimorbidity clusters: a combination of stability and dynamically

Five multimorbidity clusters were identified through LCA in this study, which were largely aligned with common patterns reported in previous studies [9, 37, 38]. Minor discrepancies in disease clusters may arise from variations in study populations, disease lists, clustering methods, and screening techniques. We conducted repeated trials and multiple calculation criteria for within-group disease characteristics to mitigate potential bias from these methodological differences. The results extends the findings of a recent study that traced the clinical trajectories of five multimorbidity clusters over 12 years among 2931 individuals [23]. While the previous study highlighted the dynamic nature of multimorbidity clusters, our findings suggest that the cluster-representative diseases remained relatively stable. Although the representative diseases showed stability within clusters, the prevalence of individual diseases within the clusters continued to reflect the dynamic nature noted in the aforementioned study. The inconsistencies may also be attributed to variations in sample sizes, disease lists and self-reported recall bias. Our study, based on a larger sample size encompassing 14 chronic diseases with high incidence among the population, mitigates the influence of clustering characteristics that may arise when numerous chronic diseases are included simultaneously.

### Additive effect of multimorbidity clusters on mortality

Consistent with previous research examining the effect of multimorbidity patterns on mortality in middle-aged and older adults [20, 23, 33], we found that the impact of different multimorbidity patterns varied. Our study revealed the persistent high-risk influence of the multisystem complex and respiratory multimorbidity patterns on individual prognosis, likely driven by cumulative disease burden and systemic vulnerabilities (e.g., overlapping inflammatory pathways or care fragmentation for complex patients). Within a 5-year follow-up period, transferring to the “Compx” or “Resp” groups significantly increased the mortality risk compared to other clusters. We also found that individual’s transitions showed differences between early and late transitions: individuals who transitioned early from the “Compx” group to the “Resp” group had the highest mortality risk among all combinations. This observation of a nine-fold mortality risk (HR:9.04) for transitions to respiratory clusters aligns with conceptual frameworks positing that respiratory complications in multisystem multimorbidity may accelerate systemic decline through pathways such as chronic inflammation or care fragmentation [36]. While Fabbri et al. only provide a syndemic perspective rather than direct empirical estimates, this theoretical context supports the plausibility of our findings. Although the small subgroup size (n = 17) limits precision, the magnitude of the hazard ratio aligns with broader evidence. For instance, studies from the China Kadoorie Biobank have consistently identified respiratory-dominant multimorbidity clusters as high-risk phenotypes for mortality [33, 34], even though these analyses focused on baseline disease patterns rather than longitudinal transitions. Groves et al. demonstrated that multimorbidity independently predicts poor outcomes in chronic respiratory diseases, reinforcing the clinical relevance of comorbid burden [35]. These lines of evidence, while methodologically distinct, collectively contextualize our observation of elevated mortality risks. To our knowledge, this is the first study to quantify the mortality risk of respiratory transitions in pre-existing multisystem multimorbidity. While direct comparative data are scarce, our results highlight a critical gap in existing literature and underscore the need for future mechanistic investigation.

Additionally, we observed volatility in the risk of death among individuals transferring to the cardiovascular multimorbidity cluster, potentially linked to acute events in cardiovascular and cerebrovascular diseases, as well as early-warning signs, timely medical intervention, and the prevalence of health management policies. By tracing longitudinal transitions between multimorbidity clusters, our findings underscore new insights into the disease accumulation hypothesis and the compounding effect of diseases on mortality, addressing a research gap concerning how dynamic shifts between multimorbidity clusters impact health outcomes, thus informing the identification and management for high-risk patients in clinical practice.

### Synergy of cumulative characteristics of chronic conditions

We found that the linear and nonlinear dose–response relationships between the number of chronic diseases and mortality rates were consistent with previous studies [39, 40]. We also established a dose-response relationship between the number of affected body systems and mortality risk, filling a gap in understanding this correlation. For individuals in the long-term “Cardm” group, mortality risk was susceptible to the cumulative growth rate of diseases during the follow-up. This may be caused by the presence and accumulation of conditions such as hypertension, dyslipidemia, and diabetes, which accelerate the cumulative incidence of cardiovascular metabolic multimorbidity [41, 42]. We also found that the mortality risk associated with the cumulative incidence rate of one or more new-onset diseases increased rapidly with a rising baseline disease burden, highlighting a nonlinear escalation of risk that may reflect both additive effects and unmeasured biological cascades [41]. This finding emphasizes the importance of disease counts and temporal factors in the cumulative effect of chronic diseases, suggesting that dynamic changes and trends can inform intervention strategies. In addition, our results indicate that the trajectory of individual transfers within the multimorbidity cluster holds higher predictive value for mortality risk than systematic growth in disease numbers, offering a reference for improved prognostic assessments and management strategies.

### Strengths and limitations

Our study holds important implications for rational prognosis and management for patients with multimorbidity. First, individual transitions between multimorbidity clusters may result from the development of key diseases of other clusters triggered by existing conditions or iatrogenic events. Therefore, tracking these transitions between multimorbidity clusters provides an efficient way to assess disease development in a large population. Second, the effects of disease count and affected systems varied across transfer trajectories, which could provide informative aid for the chronic disease population's prognostic risk management.

The study has several strengths. First, it systematically identified and tracked individual-level transitions between multimorbidity patterns in an ageing cohort longitudinally, providing a unique perspective on chronic disease progression. Second, this refined analysis aids in identifying high-risk disease combinations, providing precise guidance for clinical interventions. Third, this study revealed, for the first time, the impact of chronic disease counts and the number of affected body systems on mortality across longitudinal combinations of disease patterns, providing actionable indicators for risk stratification in clinical practice. However, our analysis could not disentangle whether these associations stem from direct disease interactions or shared upstream drivers (e.g., genetic susceptibility or socioeconomic disparities). Future studies integrating multi-omics data or causal mediation models are needed to address this gap.

Several additional limitations should also be acknowledged. First, the CHARLS questionnaire did not cover all chronic diseases prevalent in older adults in recent years. Further research is needed to understand the impact of multimorbidity caused by other prevalent chronic diseases, such as thyroid disease, gout, eye disease, etc. Second, while LCA effectively identified multimorbidity clusters, it does not directly model the longitudinal disease-disease interactions. The observed mortality differences may arise from additive effects of concurrent conditions or unmeasured biological pathways, rather than synergistic mechanisms. Such study design was to simplify the computational burden that could arise from the combination of diseases across three follow-up waves for longitudinal techniques (i.e. latent transition analysis or multistate models). Third, the data were collected at follow-up intervals rather than timely clinical diagnoses. To mitigate potential time bias, we incorporated the exact date of death recorded in the 2020 wave to specify the specific year of death or dropout. Fourth, the wide confidence intervals for certain transition trajectories (e.g., “Compx + Resp + Resp”, HR:9.04; 95% CI [3.44–23.73]) underscore the challenge of studying rare but high-risk transitions in population cohorts. While these estimates highlight potential clinical red flags, their precision is constrained by small subgroup sizes and should be interpreted with caution. Future studies with larger samples or enriched sampling of high-risk subgroups are needed to refine these associations. Fifth, while we adjusted for key lifestyle factors (smoking, alcohol use, physical activity), these variables were dichotomized (e.g., current smoker: yes/no) to prioritize model parsimony. This approach may obscure dose-dependent relationships, such as differential risks between heavy versus occasional drinkers or variations in exercise intensity/duration. Future studies integrating granular lifestyle measures (e.g., accelerometer-based physical activity, dietary biomarkers) could address these gaps. Additionally, our study prioritized transitions between multimorbidity clusters among individuals with baseline chronic diseases, rather than tracking initial transitions from a healthy state to multimorbidity. Although the “no-NCD” reference group provided mortality benchmarks, future studies should explore bidirectional transitions (e.g., healthy-to-cluster shifts) to fully capture the dynamic nature of multimorbidity progression.

## Conclusion

This study investigated the adverse health impacts of developing new chronic conditions among individuals with long-term multimorbidity by tracking their transition pathways between different multimorbidity clusters. We extend the understanding of multimorbidity dynamics by tracing the individuals’ transitions between different multimorbidity clusters, revealing the important additive effects and potentially compounding effects of disease combinations on mortality. Future research could include exploiting the biological mechanisms underlying multimorbidity patterns and exploring strategies to delay disease progression. In conclusion, this study provides a novel perspective on multimorbidity development in middle-aged and older populations, emphasizing the importance of dynamic monitoring and multi-dimensional interventions.

## Supplementary Information

Below is the link to the electronic supplementary material.

Supplementary file1 (DOCX 883 KB)

## Data Availability

No datasets were generated during the current study.
