# Supplementary material for: Individual-level transitions between chronic disease multimorbidity clusters and the risk of five-year mortality in longitudinal cohort of Chinese middle-aged and older adults
Source: Aging Clin Exp Res. 2025 Jul 9;37(1):216. doi: 10.1007/s40520-025-03078-5 (PMC12241155; doi:10.1007/s40520-025-03078-5)
Supplement: Supplementary file 1 — Supplementary file1 (DOCX 883 KB) [file 40520_2025_3078_MOESM1_ESM.docx]

**Supplemental Material**

1. **eTable 1. Chronic diseases and their corresponding systematic classifications.**
2. **eTable 2. Characteristics of healthy participants and prevalence of chronic disease in 2015.**
3. **eTable 3.** **Characteristics of included and excluded participants in 2011**
4. **eTable 4. Model fit and diagnostic criteria for competing latent class models in 2011.**
5. **eTable 5. Model fit and diagnostic criteria for competing latent class models in 2013.**
6. **eTable 6. Model fit and diagnostic criteria for competing latent class models in 2015.**
7. **eTable 7. O/E rates in the five identified multimorbidity clusters in 2011.**
8. **eTable 8. Exclusivity rates in the five identified multimorbidity clusters in 2011.**
9. **eTable 9. O/E rates in the five identified multimorbidity clusters in 2013.**
10. **eTable 10. Exclusivity rates in the five identified multimorbidity clusters in 2013.**
11. **eTable 11. O/E rates in the five identified multimorbidity clusters in 2015.**
12. **eTable 12. Exclusivity rates in the five identified multimorbidity clusters in 2015.**
13. **eFigure 1. Flowchart of the sampling process.**
14. **eFigure 2. Association between excess rate of chronic disease cumulative characteristics and all-cause mortality by multimorbidity pattern combinations.**
15. **eFigure 3. Associations between multimorbidity cluster progression and all-cause mortality risk among non-imputed datasets.**
16. **eFigure 4. Associations between multimorbidity cluster progression and all-cause mortality risk with physical activity in the fully adjusted model.**

**eTable 1. Chronic diseases and their corresponding systematic classifications**

| **Affected Body Systems** | **Chronic Disease Conditions** |
| --- | --- |
| Diseases of the Circulatory System | Hypertension, Heart problems, Stroke, Dyslipidemia |
| Diseases of the Endocrine System | Diabetes/high blood sugar |
| Cancers | Cancer or malignant tumor |
| Diseases of the Respiratory System | Chronic lung disease, Asthma |
| Musculoskeletal conditions | Arthritis or rheumatism |
| Diseases of the Digestive System | Liver disease, Stomach or other digestive disease |
| Diseases of the Genitourinary System | Kidney disease |
| Mental Health Disorders | Emotional/nervous/psychiatric problem, Memory-related disease |

**eTable 2. Characteristics of participants without the 14 NCDs from 2011 to 2015 and prevalence of chronic disease in 2015**

| **Characteristicst** | **Total** | ***P* value** |
| --- | --- | --- |
|  | N (%) | |
| participants | 1844 | - |
| Age（years） |  | ＜0.001 |
| ≤65 | 1387(75.2) |  |
| ＞65 | 457(24.8) |  |
| Sex |  | ＜0.020 |
| Male | 972(52.7) |  |
| Female | 872(47.3) |  |
| Hukou Regions |  | ＜0.001 |
| Rural | 1535(82.6) |  |
| Urban | 309(17.4) |  |
| Education |  | ＜0.001 |
| Illiterate | 454(24.6) |  |
| ≤Elementary school | 686(37.2) |  |
| ≥Middle school | 704(38.2) |  |
| Marital status |  | ＜0.001 |
| Unmarried/unpartnered | 235(12.7) |  |
| Married/partnered | 1609(87.3) |  |
| Smoking status |  | ＜0.001 |
| Current smoker | 629(34.0) |  |
| None-smoker | 1215(66.0) |  |
| Alcohol consumption |  | ＜0.001 |
| Drink alcohol last year | 725(39.3) |  |
| No alcohol last year | 1119(60.6) |  |

**eTable3：Characteristics of included and excluded participants in 2011**

| **Characteristic** | **Included Chronic^1^**  N = 7,144^2^ | **Excluded Chronic^1^**  N = 15,092^2^ | **Included No-Chronic^1^** N = 1,844^2^ | **Excluded No-Chronic^1^**  N = 1,506^2^ | **p-value**^3^ |
| --- | --- | --- | --- | --- | --- |
| Age (years) | 59.53 (9.22) | 58.14 (11.35) | 56.64 (9.11) | 57.52 (9.32) | <0.001 |
| Missing | 0 | 8,053 | 0 | 0 |  |
| Gender |  |  |  |  | <0.001 |
| Male | 3,354 (47%) | 7,237 (48%) | 972 (53%) | 771 (51%) |  |
| Female | 3,790 (53%) | 7,847 (52%) | 872 (47%) | 735 (49%) |  |
| Missing | 0 | 8 | 0 | 0 |  |
| Hukou Regions |  |  |  |  | <0.001 |
| Urban | 1,811 (25%) | 2,024 (28%) | 309 (17%) | 251 (17%) |  |
| Rural | 5,333 (75%) | 5,155 (72%) | 1,535 (83%) | 1,250 (83%) |  |
| Missing | 0 | 7,913 | 0 | 5 |  |
| Education |  |  |  |  | <0.001 |
| Illiterate | 2,060 (29%) | 3,315 (22%) | 454 (25%) | 427 (28%) |  |
| ≤Elementary school | 2,943 (41%) | 7,007 (46%) | 686 (37%) | 585 (39%) |  |
| ≥Middle school | 2,141 (30%) | 4,770 (32%) | 704 (38%) | 494 (33%) |  |
| Marital status |  |  |  |  | <0.001 |
| Married/partnered | 5963 (84%) | 6,199 (41%) | 1,609 (87%) | 1,341 (89%) |  |
| Unmarried/unpartnered | 1181 (16%) | 8,893 (59%) | 235 (12.7%) | 165 (11%) |  |
| Smoking status |  |  |  |  | <0.001 |
| None-smoker | 5270 (74%) | 4,984 (73%) | 1,215 (66%) | 942 (67%) |  |
| Current smoker | 1874 (26%) | 1,829 (27%) | 629 (34%) | 469 (33%) |  |
| Missing | 0 | 8,279 | 0 | 95 |  |
| Alcohol consumption |  |  |  |  | <0.001 |
| No alcohol last year | 4,915 (69%) | 4,838 (68%) | 1,119 (61%) | 915 (62%) |  |
| Drink alcohol last year | 2,229 (31%) | 2,259 (32%) | 725 (39%) | 567 (38%) |  |
| Missing | 0 | 7,995 | 0 | 24 |  |
| Number of  chronic conditions | 2.00  (2.00, 4.00) | 0.00  (0.00, 1.00) | 0 | 1.00  (1.00, 2.00) | <0.001 |
| ^1^Included Chronic – Individuals included in the following analyses with chronic conditions in 2011; Excluded Chronic – Individuals excluded from the CHARLS data with chronic conditions in 2011; Included No-Chronic – Individuals included in the following analyses as the non-NCD reference group; Excluded No-Chronic – Excluded individuals with no chronic conditions in 2011 but developed chronic conditions in 2013 or 2015.  ^2^Mean (SD); n (%); Median (Q1, Q3)  ^3^Kruskal-Wallis rank sum test; Pearson's Chi-squared test | | | | | |

**eTable 4. Model fit and diagnostic criteria for competing latent class models in 2011**

| **cluster** | **AIC** | | | **BIC** | | | **Chisq** | | | **Gsq** | | |
| --- | --- | --- | --- | --- | --- | --- | --- | --- | --- | --- | --- | --- |
|  | min | max | average | min | max | average | min | max | average | min | max | average |
| 3 | 65738 | 66108 | 65807 | 66040 | 66410 | 66110 | 28821 | 2222685 | 838927 | 4322 | 4692 | 4391 |
| 4 | 65156 | 65881 | 65335 | 65562 | 66287 | 65741 | 26156 | 1089737 | 252451 | 3711 | 4435 | 3889 |
| 5 | 64733 | 65305 | 64896 | 65241 | 65814 | 65405 | 28004 | 666669 | 78464 | 3257 | 3829 | 3420 |
| 6 | 64455 | 65266 | 64566 | 65067 | 65878 | 65178 | 25022 | 85894 | 55988 | 2949 | 3760 | 3060 |
| 7 | 64243 | 64994 | 64304 | 64958 | 65709 | 65019 | 20758 | 82632 | 44583 | 2707 | 3458 | 2768 |
| 8 | 64105 | 64960 | 64204 | 64923 | 65778 | 65022 | 23339 | 84348 | 52673 | 2539 | 3394 | 2638 |
| 9 | 64006 | 64358 | 64082 | 64928 | 65279 | 65003 | 23930 | 343420 | 46740 | 2410 | 2762 | 2486 |
| 10 | 63887 | 64100 | 64000 | 64911 | 65124 | 65024 | 16026 | 126636 | 35350 | 2261 | 2474 | 2374 |

**eTable 5. Model fit and diagnostic criteria for competing latent class models in 2013**

| **cluster** | **AIC** | | | **BIC** | | | **Chisq** | | | **Gsq** | | |
| --- | --- | --- | --- | --- | --- | --- | --- | --- | --- | --- | --- | --- |
|  | min | max | average | min | max | average | min | max | average | min | max | average |
| 3 | 70224 | 70635 | 70270 | 70527 | 70938 | 70573 | 21953 | 7594775 | 784554 | 4370 | 4781 | 4416 |
| 4 | 69734 | 70278 | 69770 | 70140 | 70683 | 70176 | 20707 | 1496753 | 157081 | 3850 | 4393 | 3885 |
| 5 | 69246 | 69706 | 69353 | 69754 | 70215 | 69862 | 21010 | 996255 | 47676 | 3331 | 3791 | 3439 |
| 6 | 69054 | 69501 | 69138 | 69666 | 70113 | 69750 | 19692 | 44282 | 37290 | 3109 | 3557 | 3193 |
| 7 | 68879 | 69446 | 68926 | 69594 | 70161 | 69641 | 19605 | 44189 | 29914 | 2905 | 3472 | 2952 |
| 8 | 68796 | 69027 | 68818 | 69614 | 69845 | 69636 | 19604 | 54136 | 28678 | 2792 | 3022 | 2813 |
| 9 | 68675 | 69393 | 68737 | 69596 | 70314 | 69658 | 10961 | 45905 | 28348 | 2640 | 3358 | 2702 |
| 10 | 68604 | 68772 | 68668 | 69628 | 69797 | 69692 | 14514 | 58205 | 27746 | 2539 | 2708 | 2603 |

**eTable 6. Model fit and diagnostic criteria for competing latent class models in 2015.**

| **cluster** | **AIC** | | | **BIC** | | | **Chisq** | | | **Gsq** | | |
| --- | --- | --- | --- | --- | --- | --- | --- | --- | --- | --- | --- | --- |
|  | min | max | average | min | max | average | min | max | average | min | max | average |
| 3 | 79948 | 80005 | 79973 | 80250 | 80307 | 80275 | 35367 | 268357 | 165841 | 4678 | 4735 | 4703 |
| 4 | 79389 | 79818 | 79393 | 79795 | 80223 | 79799 | 26215 | 498291 | 30935 | 4089 | 4518 | 4094 |
| 5 | 79220 | 79319 | 79237 | 79729 | 79828 | 79746 | 22929 | 28777 | 25638 | 3891 | 3989 | 3907 |
| 6 | 79076 | 79163 | 79106 | 79688 | 79775 | 79718 | 21995 | 35811 | 25500 | 3716 | 3803 | 3746 |
| 7 | 78957 | 79084 | 79002 | 79672 | 79799 | 79717 | 19071 | 36581 | 24685 | 3567 | 3694 | 3612 |
| 8 | 78855 | 79059 | 78914 | 79673 | 79877 | 79732 | 15369 | 40035 | 23969 | 3435 | 3640 | 3494 |
| 9 | 78762 | 79010 | 78806 | 79683 | 79931 | 79728 | 15215 | 35698 | 21364 | 3312 | 3560 | 3357 |
| 10 | 78706 | 78993 | 78752 | 79730 | 80018 | 79777 | 14656 | 34878 | 20132 | 3226 | 3513 | 3273 |

**eTable 7. O/E rates in the five identified multimorbidity clusters in 2011**

| **chronic disease condition** | **Resp** | **Compx** | **Arth** | **Digst** | **Cardm** |
| --- | --- | --- | --- | --- | --- |
| Hypertension | 0.50 | 2.03 | 0.51 | 0.38 | 1.77 |
| Diabetes/high blood sugar | 0.20 | 2.99 | 0.17 | 0.23 | 2.03 |
| Cancer or malignant tumor | 0.28 | 3.94 | 0.00 | 0.70 | 1.56 |
| Chronic lung disease | 6.57 | 2.28 | 0.00 | 0.04 | 0.03 |
| Heart problems | 0.71 | 4.26 | 0.28 | 0.45 | 1.29 |
| Stroke | 0.30 | 3.60 | 0.38 | 0.23 | 1.69 |
| Emotional/nervous/psychiatric problem | 0.76 | 2.19 | 0.52 | 0.54 | 1.51 |
| Arthritis or rheumatism | 0.69 | 1.66 | 2.01 | 0.96 | 0.20 |
| Dyslipidemia | 0.12 | 3.44 | 0.21 | 0.42 | 1.79 |
| Liver disease | 0.80 | 2.55 | 0.21 | 1.23 | 1.15 |
| Kidney disease | 0.90 | 2.58 | 0.61 | 1.05 | 0.93 |
| Stomach or other digestive disease | 0.68 | 2.06 | 0.00 | 3.12 | 0.11 |
| Asthma | 5.73 | 2.37 | 0.35 | 0.09 | 0.03 |
| Memory-related disease | 0.34 | 4.17 | 0.38 | 0.26 | 1.51 |

**Note.** Participants were divided into five patterns based on conditions at baseline: osteoarticular multimorbidity (“Arth”), cardiometabolic multimorbidity (“Cardm”)， multisystem complex pattern (“Compx”)， digestive multimorbidity (“Digst”)， and respiratory multimorbidity (“Resp”).

**eTable 8. Exclusivity rates in the five identified multimorbidity clusters in 2011**

| **chronic disease condition** | **Resp** | **Compx** | **Arth** | **Digst** | **Cardm** |
| --- | --- | --- | --- | --- | --- |
| Hypertension | 0.06 | 0.16 | 0.13 | 0.09 | 0.57 |
| Diabetes/high blood sugar | 0.02 | 0.23 | 0.04 | 0.05 | 0.65 |
| Cancer or malignant tumor | 0.03 | 0.30 | 0.00 | 0.16 | 0.50 |
| Chronic lung disease | 0.80 | 0.17 | 0.00 | 0.01 | 0.01 |
| Heart problems | 0.09 | 0.33 | 0.07 | 0.10 | 0.41 |
| Stroke | 0.04 | 0.28 | 0.09 | 0.05 | 0.54 |
| Emotional/nervous/psychiatric problem | 0.09 | 0.17 | 0.13 | 0.12 | 0.48 |
| Arthritis or rheumatism | 0.09 | 0.13 | 0.50 | 0.22 | 0.06 |
| Dyslipidemia | 0.02 | 0.26 | 0.05 | 0.10 | 0.57 |
| Liver disease | 0.10 | 0.20 | 0.05 | 0.28 | 0.37 |
| Kidney disease | 0.11 | 0.20 | 0.15 | 0.24 | 0.30 |
| Stomach or other digestive disease | 0.08 | 0.16 | 0.00 | 0.72 | 0.04 |
| Asthma | 0.70 | 0.18 | 0.09 | 0.02 | 0.01 |
| Memory-related disease | 0.04 | 0.32 | 0.09 | 0.06 | 0.49 |

**Note:** Participants were divided into five patterns based on conditions at baseline: osteoarticular multimorbidity (“Arth”), cardiometabolic multimorbidity (“Cardm”), multisystem complex pattern (“Compx”), digestive multimorbidity (“Digst”), and respiratory multimorbidity (“Resp”).

**eTable 9. O/E rates in the five identified multimorbidity clusters in 2013**

| **chronic disease condition** | **Arth** | **Compx** | **Digst** | **Resp** | **Cardm** |
| --- | --- | --- | --- | --- | --- |
| Hypertension | 0.52 | 1.87 | 0.40 | 0.55 | 1.72 |
| Diabetes/high blood sugar | 0.22 | 2.76 | 0.24 | 0.19 | 1.94 |
| Cancer or malignant tumor | 0.13 | 4.47 | 0.59 | 0.08 | 1.30 |
| Chronic lung disease | 0.00 | 2.08 | 0.00 | 5.93 | 0.07 |
| Heart problems | 0.26 | 3.61 | 0.47 | 0.71 | 1.27 |
| Stroke | 0.37 | 3.27 | 0.24 | 0.14 | 1.70 |
| Emotional/nervous/psychiatric problem | 0.45 | 2.22 | 0.65 | 0.79 | 1.39 |
| Arthritis or rheumatism | 1.95 | 1.55 | 0.96 | 0.78 | 0.25 |
| Dyslipidemia | 0.23 | 3.15 | 0.39 | 0.21 | 1.70 |
| Liver disease | 0.23 | 2.97 | 1.04 | 0.89 | 1.00 |
| Kidney disease | 0.65 | 2.84 | 0.96 | 0.87 | 0.79 |
| Stomach or other digestive disease | 0.00 | 2.04 | 2.93 | 0.79 | 0.13 |
| Asthma | 0.26 | 2.26 | 0.00 | 5.37 | 0.06 |
| Memory-related disease | 0.35 | 3.37 | 0.19 | 0.37 | 1.62 |

**Note:** Participants were divided into five patterns based on conditions at baseline: osteoarticular multimorbidity (“Arth”), cardiometabolic multimorbidity (“Cardm”), multisystem complex pattern (“Compx”), digestive multimorbidity (“Digst”), and respiratory multimorbidity (“Resp”).

**eTable 10. Exclusivity rates in the five identified multimorbidity clusters in 2013**

| **chronic disease condition** | **Arth** | **Compx** | **Digst** | **Resp** | **Cardm** |
| --- | --- | --- | --- | --- | --- |
| Hypertension | 0.12 | 0.18 | 0.09 | 0.07 | 0.54 |
| Diabetes/high blood sugar | 0.05 | 0.26 | 0.05 | 0.03 | 0.61 |
| Cancer or malignant tumor | 0.03 | 0.42 | 0.13 | 0.01 | 0.41 |
| Chronic lung disease | 0.00 | 0.19 | 0.00 | 0.78 | 0.02 |
| Heart problems | 0.06 | 0.34 | 0.11 | 0.09 | 0.40 |
| Stroke | 0.09 | 0.31 | 0.05 | 0.02 | 0.54 |
| Emotional/nervous/psychiatric problem | 0.10 | 0.21 | 0.15 | 0.10 | 0.44 |
| Arthritis or rheumatism | 0.45 | 0.15 | 0.22 | 0.10 | 0.08 |
| Dyslipidemia | 0.05 | 0.29 | 0.09 | 0.03 | 0.53 |
| Liver disease | 0.05 | 0.28 | 0.24 | 0.12 | 0.31 |
| Kidney disease | 0.15 | 0.27 | 0.22 | 0.11 | 0.25 |
| Stomach or other digestive disease | 0.00 | 0.19 | 0.66 | 0.10 | 0.04 |
| Asthma | 0.06 | 0.21 | 0.00 | 0.71 | 0.02 |
| Memory-related disease | 0.08 | 0.32 | 0.04 | 0.05 | 0.51 |

**Note:** Participants were divided into five patterns based on conditions at baseline: osteoarticular multimorbidity (“Arth”), cardiometabolic multimorbidity (“Cardm”), multisystem complex pattern (“Compx”), digestive multimorbidity (“Digst”), and respiratory multimorbidity (“Resp”).

**eTable 11. O/E rates in the five identified multimorbidity clusters in 2015**

| **chronic disease condition** | **Compx** | **Resp** | **Digst** | **Arth** | **Cardm** |
| --- | --- | --- | --- | --- | --- |
| Hypertension | 1.68 | 0.65 | 0.45 | 0.57 | 1.71 |
| Diabetes/high blood sugar | 2.64 | 0.22 | 0.28 | 0.25 | 1.93 |
| Cancer or malignant tumor | 2.55 | 0.17 | 1.02 | 0.35 | 1.21 |
| Chronic lung disease | 2.36 | 4.69 | 0.64 | 0.02 | 0.18 |
| Heart problems | 3.01 | 0.97 | 0.64 | 0.18 | 1.24 |
| Stroke | 2.93 | 0.25 | 0.08 | 0.63 | 1.82 |
| Emotional/nervous/psychiatric problem | 2.86 | 0.81 | 1.14 | 0.72 | 0.55 |
| Arthritis or rheumatism | 1.45 | 0.79 | 1.05 | 1.68 | 0.51 |
| Dyslipidemia | 2.56 | 0.26 | 0.43 | 0.00 | 1.93 |
| Liver disease | 3.22 | 0.82 | 1.26 | 0.25 | 0.60 |
| Kidney disease | 3.28 | 0.68 | 1.15 | 0.60 | 0.54 |
| Stomach or other digestive disease | 1.86 | 0.47 | 2.22 | 0.09 | 0.29 |
| Asthma | 2.87 | 5.18 | 0.06 | 0.49 | 0.15 |
| Memory-related disease | 3.48 | 0.48 | 0.18 | 0.58 | 1.50 |

**Note:** Participants were divided into five patterns based on conditions at baseline: osteoarticular multimorbidity (“Arth”), cardiometabolic multimorbidity (“Cardm”), multisystem complex pattern (“Compx”), digestive multimorbidity (“Digst”), and respiratory multimorbidity (“Resp”).

**eTable 12. Exclusivity rates in the five identified multimorbidity clusters in 2015**

| **chronic disease condition** | **Resp** | **Compx** | **Arth** | **Digst** | **Cardm** |
| --- | --- | --- | --- | --- | --- |
| Hypertension | 0.15 | 0.07 | 0.14 | 0.10 | 0.54 |
| Diabetes/high blood sugar | 0.24 | 0.03 | 0.08 | 0.05 | 0.61 |
| Cancer or malignant tumor | 0.23 | 0.02 | 0.31 | 0.06 | 0.38 |
| Chronic lung disease | 0.22 | 0.53 | 0.19 | 0.00 | 0.06 |
| Heart problems | 0.27 | 0.11 | 0.19 | 0.03 | 0.39 |
| Stroke | 0.27 | 0.03 | 0.02 | 0.11 | 0.57 |
| Emotional/nervous/psychiatric problem | 0.26 | 0.09 | 0.34 | 0.13 | 0.17 |
| Arthritis or rheumatism | 0.13 | 0.09 | 0.32 | 0.30 | 0.16 |
| Dyslipidemia | 0.23 | 0.03 | 0.13 | 0.00 | 0.61 |
| Liver disease | 0.29 | 0.09 | 0.38 | 0.05 | 0.19 |
| Kidney disease | 0.30 | 0.08 | 0.35 | 0.11 | 0.17 |
| Stomach or other digestive disease | 0.17 | 0.05 | 0.67 | 0.02 | 0.09 |
| Asthma | 0.26 | 0.59 | 0.02 | 0.09 | 0.05 |
| Memory-related disease | 0.32 | 0.05 | 0.05 | 0.10 | 0.47 |

**Note:** Participants were divided into five patterns based on conditions at baseline: osteoarticular multimorbidity (“Arth”), cardiometabolic multimorbidity (“Cardm”), multisystem complex pattern (“Compx”), digestive multimorbidity (“Digst”), and respiratory multimorbidity (“Resp”).

**eFigure 1. Flowchart of the sampling process**


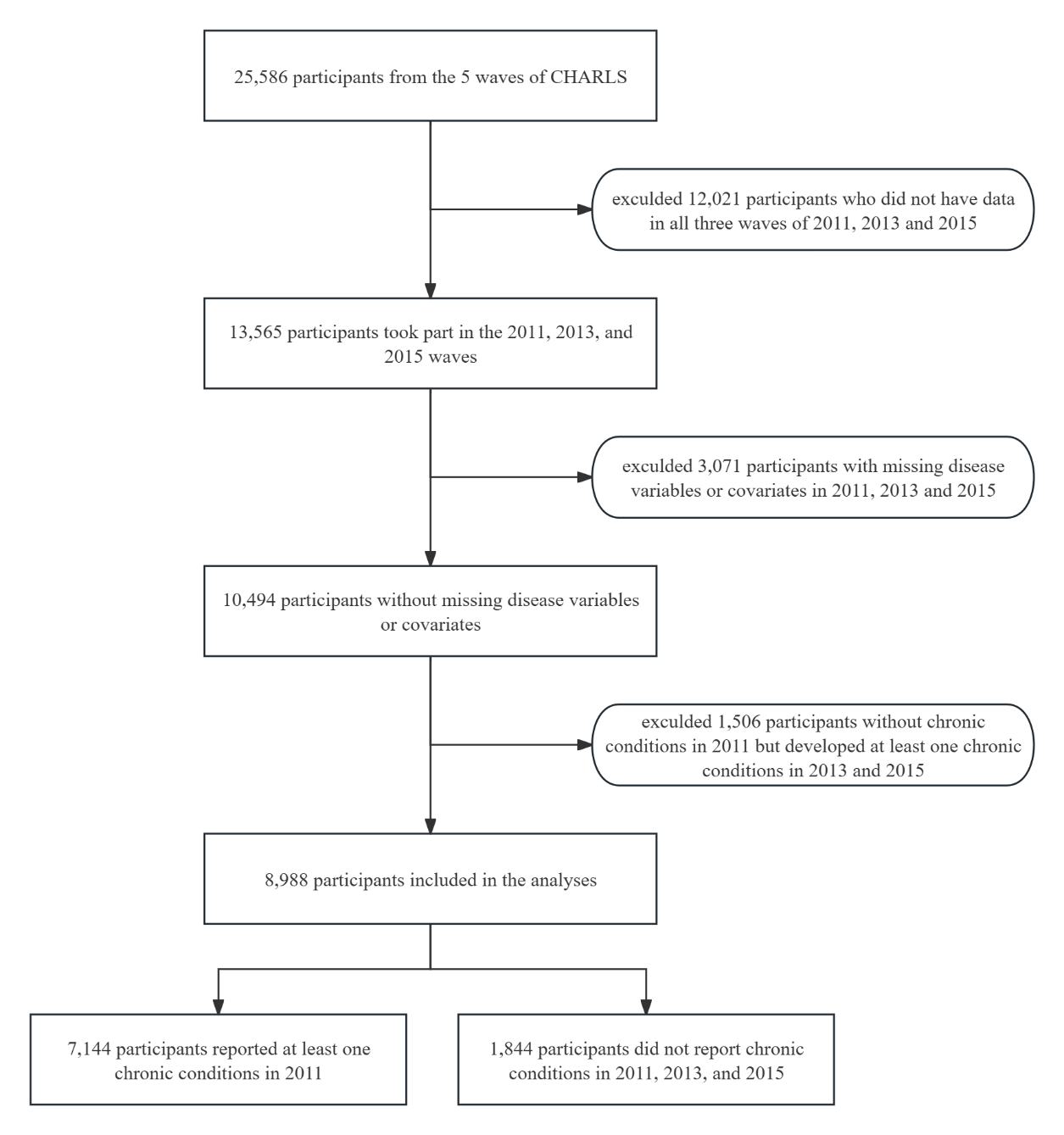


**eFigure 2. Association between excess rate of chronic disease cumulative characteristics and all-cause mortality by multimorbidity pattern combinations**


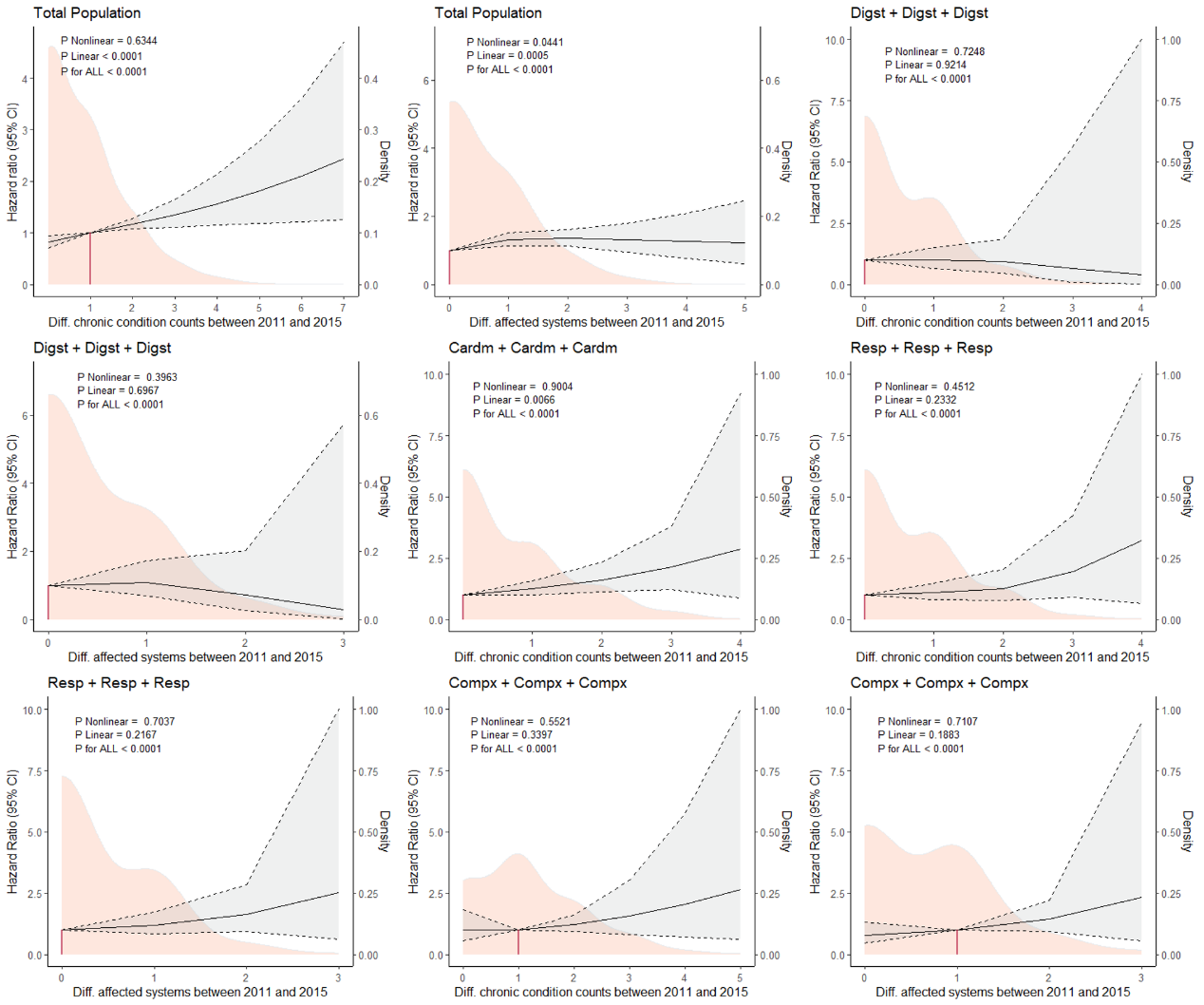


**Note:** Participants were divided into five patterns based on conditions at baseline: osteoarticular multimorbidity (“Arth”), cardiometabolic multimorbidity (“Cardm”), multisystem complex pattern (“Compx”), digestive multimorbidity (“Digst”), and respiratory multimorbidity (“Resp”). Solid black line: Estimated hazard ratio (HR) with 95% confidence interval (CI) shaded in gray. Orange density curve: Distribution of the predictor variable in the study population. Fully-adjusted model was adjusted for all controllers. *P＜0.1, **P＜0.05, ***P＜0.001.

**eFigure 3. Associations between multimorbidity cluster progression and all-cause mortality risk among non-imputed datasets**


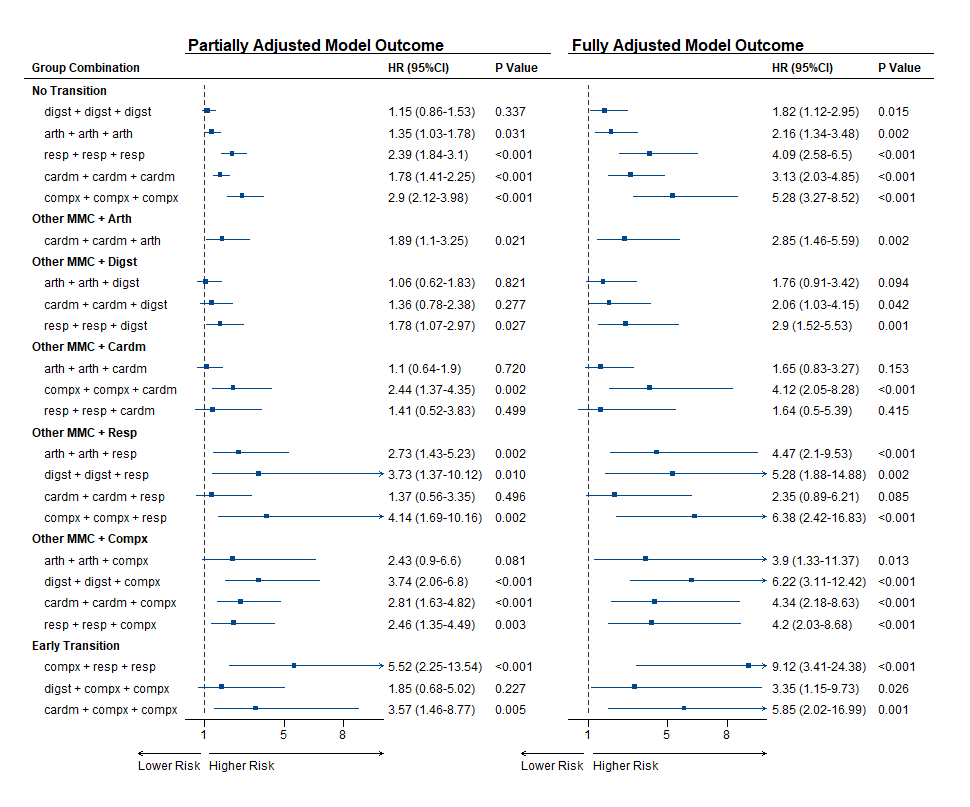


**Note:** Participants were divided into five patterns based on conditions at baseline: osteoarticular multimorbidity (“Arth”), cardiometabolic multimorbidity (“Cardm”), multisystem complex pattern (“Compx”), digestive multimorbidity (“Digst”), and respiratory multimorbidity (“Resp”).


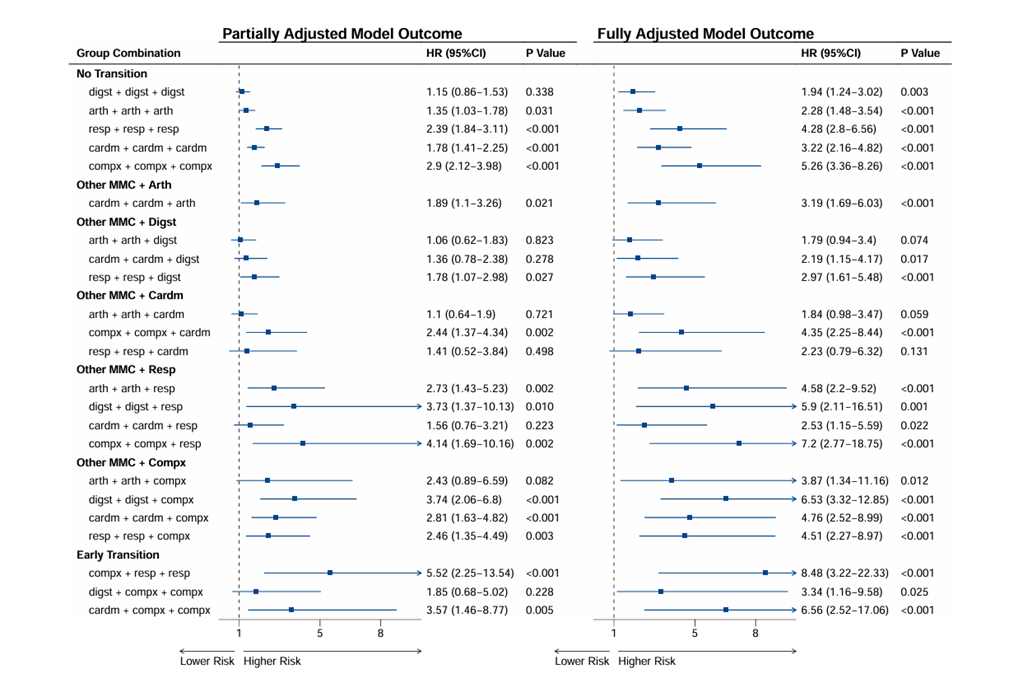
**eFigure 4. Associations between multimorbidity cluster progression and all-cause mortality risk with physical activity in the fully adjusted model.**

**Note:** Participants were divided into five patterns based on conditions at baseline: osteoarticular multimorbidity (“Arth”), cardiometabolic multimorbidity (“Cardm”), multisystem complex pattern (“Compx”), digestive multimorbidity (“Digst”), and respiratory multimorbidity (“Resp”).
